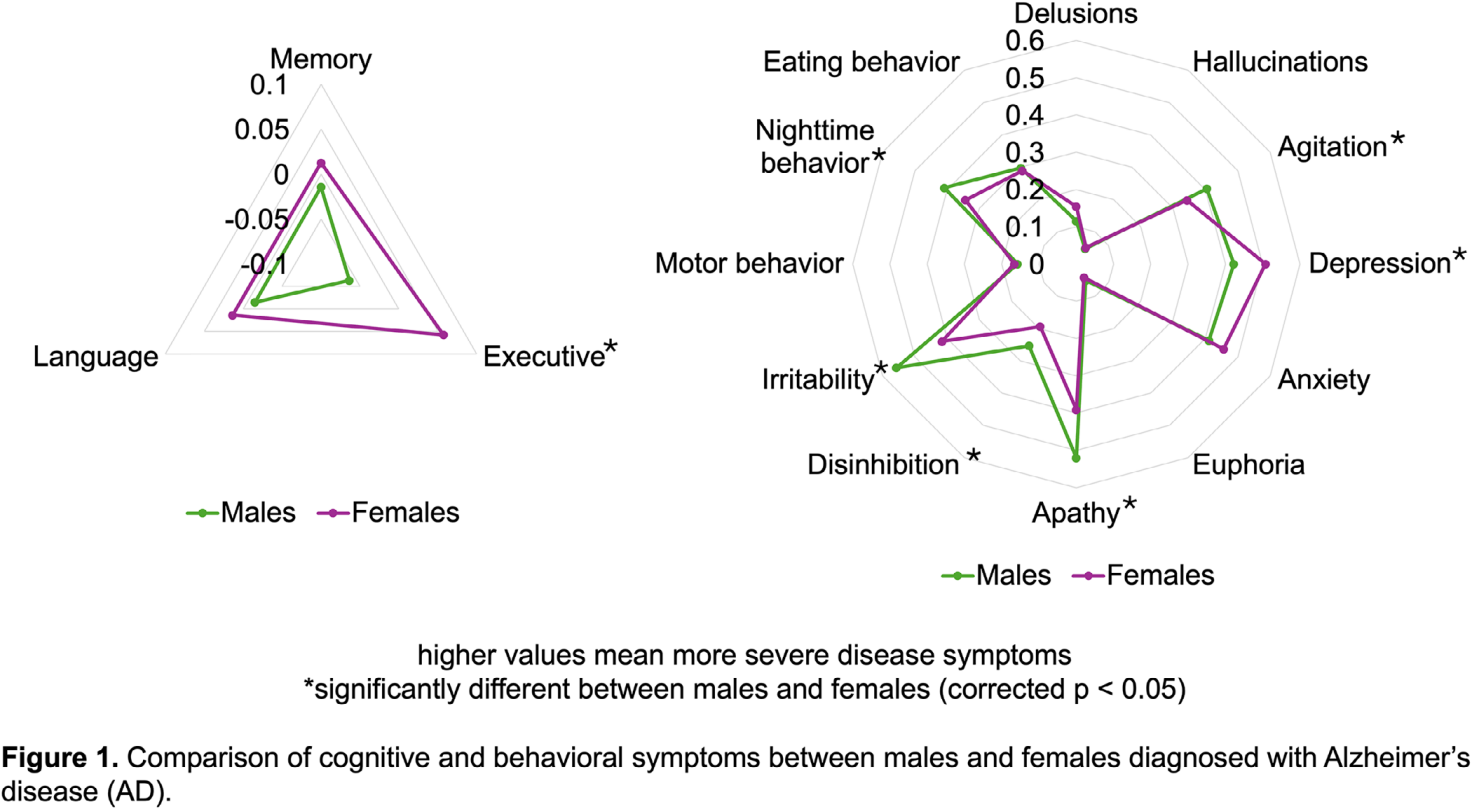# Do males and females show distinct symptoms in Alzheimer's disease and behavioral variant frontotemporal dementia?

**DOI:** 10.1002/alz70857_104722

**Published:** 2025-12-25

**Authors:** Xulin Liu, Sterre C.M. de Boer, Carmela Tartaglia

**Affiliations:** ^1^ University Health Network, Toronto, ON, Canada; ^2^ University of Toronto, Toronto, ON, Canada; ^3^ Alzheimer Center Amsterdam, Neurology, Vrije Universiteit Amsterdam, Amsterdam UMC location VUmc, Amsterdam, Noord‐Holland, Netherlands; ^4^ Amsterdam Neuroscience, Neurodegeneration, Amsterdam, Noord‐Holland, Netherlands; ^5^ The University of Sydney, School of Psychology and Brain & Mind Centre, Sydney, NSW, Australia

## Abstract

**Background:**

An imbalanced sex ratio has been reported in sporadic behavioral variant frontotemporal dementia (bvFTD), along with differences in clinical symptoms between sporadic and genetic cases, particularly in females. We hypothesize that some females without genetic mutations may be misdiagnosed with Alzheimer's disease (AD) due to atypical bvFTD symptom presentation.

**Method:**

We examined participants with probable or possible AD from the National Alzheimer's Coordinating Center (NACC) dataset (*N* = 11,286, 5,923 females and 5,363 males). Sex differences in neuropsychiatric symptoms were assessed using the Neuropsychiatric Inventory Questionnaire (NPI‐Q), and cognitive function was evaluated across major domains using standardized cognitive tests. Subgroup analyses were conducted for early‐onset AD (age < 65 years, n = 1,465) and late‐onset AD (*n* = 9,821). Analyses were corrected for age, disease severity, and education years.

**Result:**

Females exhibited more severe executive deficits (F (4, 11281) = 932.8, |β| = 0.084, *p* < 0.001) and depression (F (4, 11281) = 112.4, |β| = 0.080, *p* < 0.001) compared to males (Figure 1); while males exhibited more severe irritability (F (4, 11281) = 134.9, |β| = 0.15, *p* < 0.001), apathy (F (4, 11281) = 474.4, |β| = 0.14, *p* < 0.001), disinhibition (F (4, 11281) = 174.4, |β| = 0.062, *p* < 0.001), agitation (F (4, 11281) = 199, |β| = 0.067, *p* < 0.001) and nighttime behavioral disturbances (F (4, 11281) = 92.28, |β| = 0.072, *p* < 0.001). Males with early‐onset AD were particularly more severe in symptoms of motor behavior disturbances, disinhibition and delusions.

**Conclusion:**

These findings reveal sex‐related differences in the presentation of behavioral and cognitive symptoms in AD, with females displaying more executive deficits, and males exhibiting more behavioral disturbances. These differences may contribute to diagnostic misclassification, particularly in females with atypical bvFTD‐like symptoms. Improved recognition of sex‐related symptom variability is crucial for enhancing diagnostic accuracy and ensuring appropriate clinical management.